# Assessing heavy metal and physiochemical pollution load of Danro River and its management using floating bed remediation

**DOI:** 10.1038/s41598-024-60511-x

**Published:** 2024-04-30

**Authors:** Aditi Majumdar, Kirti Avishek

**Affiliations:** Present Address: Department of Civil and Environmental Engineering, Birla Institute of Technology Mesra, Ranchi, 835215 Jharkhand India

**Keywords:** Danro River, Physicochemical variables, Principal component analysis, Pollution index, Heavy metals, Environmental sciences, Hydrology, Limnology

## Abstract

River Danro in Garhwa (India) plays a vital role as a significant source of surface water and a crucial tributary of the North Koel River, ultimately joining the Ganga River Basin. Serving both urban-industrial and rural areas, the region faces challenges, including sand mining near Belchampa Ghat. This study aimed to assess physicochemical and heavy metals pollution at nine sampling locations, utilizing the Overall Index of Pollution (OIP), Nemerow Pollution Index (NPI), and Heavy Metal Pollution Index (HPI). OIP values indicated excellent surface water quality (0.71) in non-monsoon and slight pollution (6.28) in monsoon. NPI ranged from 0.10 to 1.74 in non-monsoon and from 0.22 (clean) to 27.15 (heavily polluted) in monsoon. HPI results suggested groundwater contamination, particularly by lead. Principal component analysis (PCA) and geospatial mapping showed similar outcomes, highlighting the influence of adjacent land use on water quality. Recognizing the significance of the Danro River in sustaining life, livelihoods, and economic growth, the study recommends implementing measures like floating bed remediation and regulatory actions for effective river management. The study acknowledges weaknesses in the current practical assessment methods for water contamination. These weaknesses make it difficult to put plans for cleaning up and controlling contamination into action. Because of this, future research on developing new in-place remediation techniques should focus on creating better ways to measure how effective the cleanup is.

## Introduction

The importance of water in supporting life is of utmost importance, and the quality of water holds equal significance^1–5^. Water has a distinct capability to dissolve and transport different substances in suspension, rendering it vulnerable to contamination^6^. Beyond fulfilling industrial and agricultural requirements, rivers also play a crucial role in providing water for essential daily activities like drinking, bathing, and washing. Simultaneously, they serve as a receptacle for substantial amounts of waste from industries, domestic sewage, and agriculture, rendering rivers highly vulnerable as a form of surface water^7–9^.

Prior research has illustrated that both human-made and natural elements, specifically land use/land cover (LULC), landscape composition^10,11^, hydro-climatology^12^, and topography, are crucial factors in influencing river water quality^11,13–15^. Human-induced alterations in land use emerge as a significant catalyst in shaping the characteristics of river water quality^16–18^. For instance, runoff from construction and agricultural areas often contains excessive nutrients like nitrogen and phosphorus, leading to nutrient pollution in rivers and subsequently deteriorating aquatic ecosystems and water quality^19–21^.

The study area was selected due to its status associated with illegal sand mining resulting in river water quality and habitat destruction^22^. In the monsoon period, the Danro River transports a notable quantity of sediments, comprising rocks, gravel, and sand. Field observations reveal that the accumulation of these materials results in sediment dunes, continuously altering morphological characteristics and causing bank erosion^23,24^. At the same time, the quality of the river water has declined to the extent of experiencing eutrophication, a condition linked to non-point source pollution originating from agricultural activities and the release of domestic and industrial sewage^25–28^. Belchampa Ghat, situated along the riverbanks, contends with widespread illegal sand mining, provoking severe environmental consequences. The unregulated activities not only degrade water quality but also compromise nearby infrastructure's structural integrity, heightening the risk of flooding. This unchecked exploitation not only endangers the delicate ecosystem but also imperils the livelihoods of local communities, necessitating immediate intervention to mitigate the escalating repercussions.

The assessment of water quality in the past has involved the investigation of several physicochemical and biological elements, including nitrate, temperature, total phosphate (TP), turbidity, pH, dissolved oxygen (DO), faecal coliform (FC), and total solids (TS)^29^. Various organizations have proposed and adopted mathematical and statistical methods using different indices for evaluating these parameters^30,31^. Among these metrics, the Overall Index of Pollution (OIP) is especially valuable for understanding the water quality status of surface water sources, particularly in the context of India^32^. Another noteworthy pollution index is the Nemerow Pollution Index (NPI), created by Nemerow and Sumitomo, which, as suggested by Refs.^33,34^, offers insights into the parameters responsible for changes in water quality status. Additionally, the presence of heavy metals poses a significant threat to human life even at low concentrations^35–39^. Water contamination by heavy metals occurs due to a blend of natural processes such as the chemical weathering of minerals and soil leaching, along with human activities including the release of industrial and domestic effluents, landfill leachate, water runoff, urban stormwater, and mining. Numerous investigations^40–46^ have emphasized that the contamination of water with heavy metals can result in various health issues, including tumours, head congestion, and muscular edema. An extensively used approach for gauging pollution levels in water bodies involves the computation of the heavy metal pollution index, offering insights into the origins of heavy metals^47,48^.

Monitoring water quality stands as a top priority in environmental protection policies^49,50^. Numerous researchers have already concentrated on assessing the current physico-chemical characteristics of water^51–55^. Despite the Indian government's initiation of various programs and action plans, involving substantial financial investments, to mitigate pollution levels in the Ganges and its tributaries, positive outcomes remain elusive^56–60^. Notably, the Ganges Action Plan (GAP), launched in 1986 with a budget of INR 9017.1 million, was deemed a failure and discontinued in 2000. The follow-up endeavour, Namami Gange, launched by the National Democratic Alliance government in mid-May 2015 with a budget of INR 22,000 million and an expectation of substantial improvement by 2019, also failed to achieve success even after five years^61–64^. Hence, there is a pressing need for additional case studies to conduct micro-level analyses, identifying local factors influencing water quality. This approach is essential for ensuring the efficient management and protection of aquatic life^65,66^.

Based on the aforementioned considerations, the primary aim of this study is to establish the correlation between the water quality of the Danro River and the land uses within its sphere of influence. Additional objectives include assessing the river's water quality using physical and chemical parameters at various points, employing the Overall Index of Pollution (OIP), Nemerow Pollution Index (NPI), and Heavy Metal Pollution Index (HPI). The investigation aims to establish connections between water quality variables and Land Use/Land Cover (LULC) by employing statistical tools that correlate the index and its variables with distinct land uses. Therefore, this research presents an innovative study to examine the association between land use and water quality in the vulnerable Danro River in Garhwa district, affected by unauthorized sand mining activities. The primary emphasis is on a specific section to tackle pollution concerns from a broader landscape viewpoint, evaluating the viability and relevance of such investigations on a smaller scale before scaling up to cover the entire basin. This research innovatively calculates the percentage of various land uses within a 10 km radius of the river bank along the entire Garhwa stretch, offering suggestions for mitigating pollution loads.

## Materials and methods

### Study area

**Figure 1:**
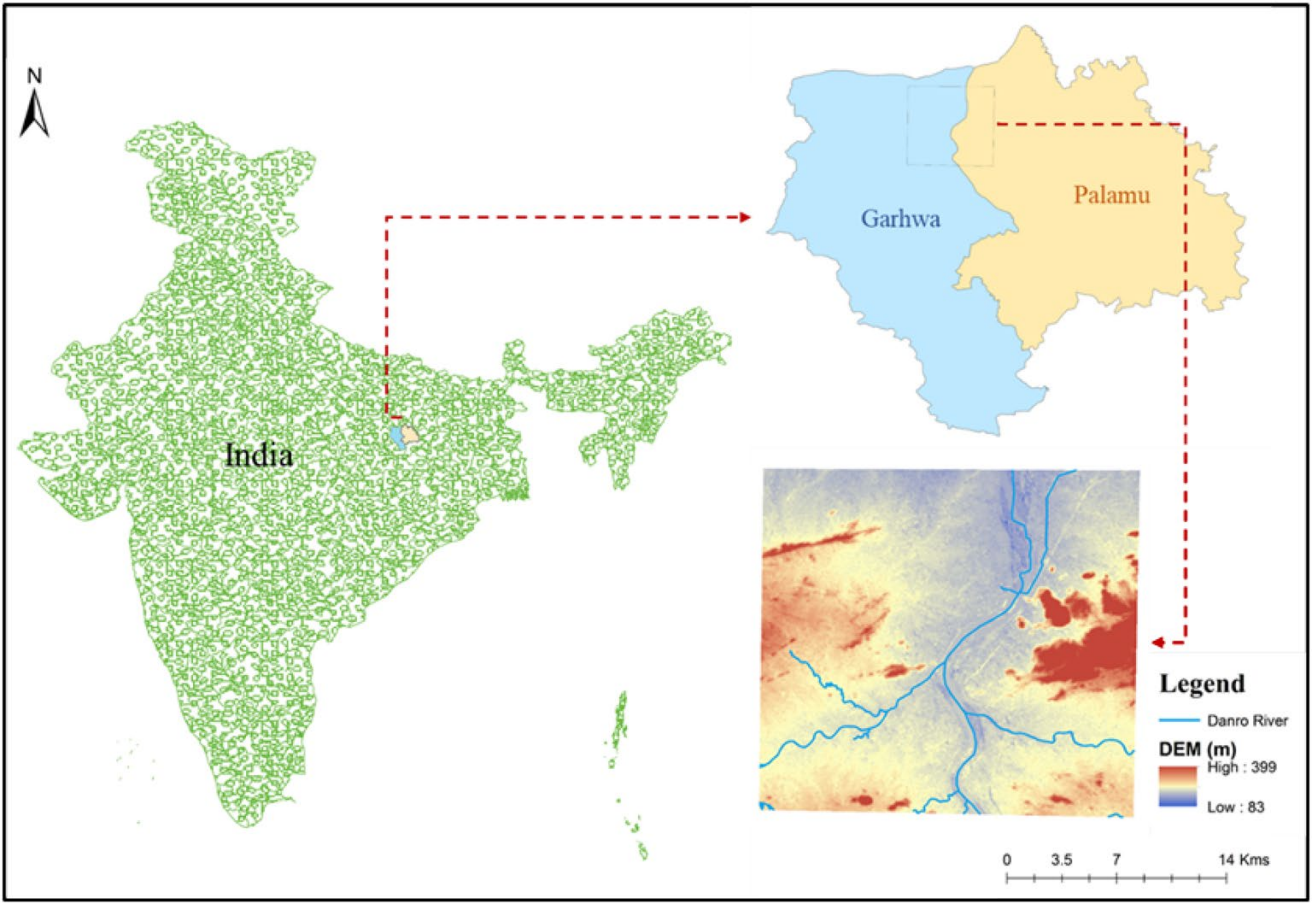
Map of the study area depicting the elevation.

**Figure 2:**
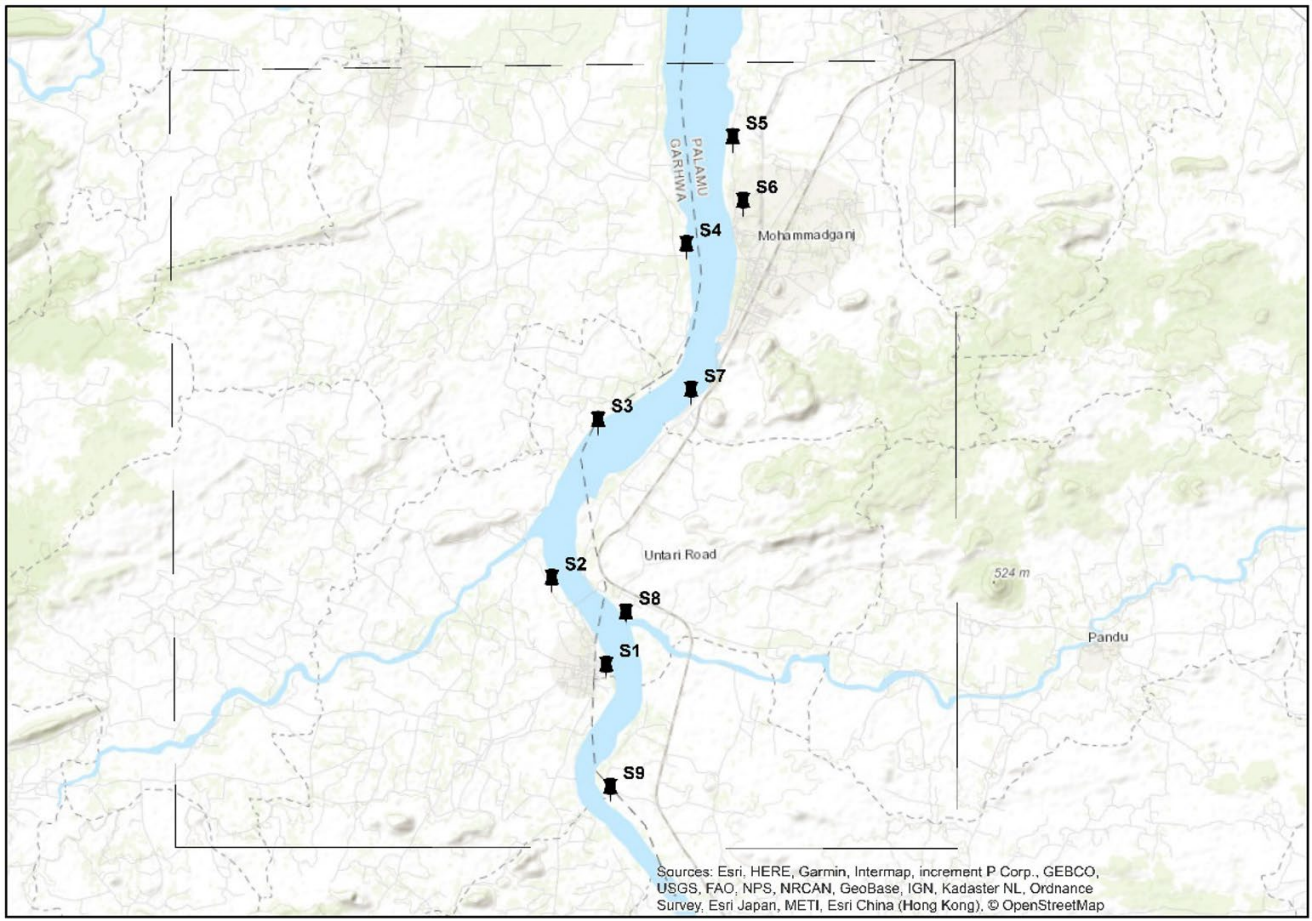
Satellite image of selected sampling sites.

The present study was carried out in the Danro River Basin, located in Garhwa City covering an area of 520.40 km^2^. The river is located between 23°60’ and 24°39’ N latitude and 83°22’ and 84°00’ longitude (Fig. 1). The local population rely on the Danro River as their primary source of water. The study area includes a peri-urban area, having an elevation range from 226 to 608 m. Danro River Basin, which flows into the North Koel River which ultimately joins the Ganga River Basin is covered in undulating terrain with a modest slope. The basin receives high rainfall (1500 mm/year) and drains quickly^67^. Land use is diverse with forests (811.4 km^2^) and agriculture (1617.5 km^2^ + 1693 km^2^ cultivable). The major soil types in the basin include Ultisols, which are characterized by acidic, nutrient-poor soils^68^. The climate is seasonal with a rainy season (Nov-Mar). Most of the basin is arid but the north is temperate and the south is tropical savannah^67^. The basin is economically important (agriculture, tourism, fishing, forestry, mining, transportation) but faces challenges like heavy metal pollution, decreased water table, soil erosion, and declining sand deposits due to mining. In this study, a total of nine sampling locations (S1–S9) were chosen (refer to Fig. 2). The geographic coordinates, notable landmarks, and delineation of these sampling sites are detailed in Table 1.

**Table 1:** Location of the sampling sites and their geo-coordinates of Danro River at Garhwa district.

Sampling site	Demarcation	Landmark of sampling zone	Geo-coordinates of sampling sites		Elevation
Site-1	S1	Sahaspura	24°19′25.89"	83°49′21.81"	172 m
Site-2	S2	Pirthi Chak	24°20′49.63"	83°48′27.06"	163 m
Site-3	S3	Burhi Khar	24°23′19.02"	83°49′18.60"	161 m
Site-4	S4	Jharha	24°26′4.47"	83°50′54.01"	166 m
Site-5	S5	Ranadih	24°27′45.41"	83°51′44.24"	154 m
Site-6	S6	Birdhawar	24°26′44.38"	83°51′53.48"	155 m
Site-7	S7	Bheem Chulha	24°23′45.70"	83°50′55.65"	165 m
Site-8	S8	Unnari Road	24°20′15.45"	83°49′43.19"	167 m
Site-9	S9	Joga	24°17′29.67"	83°49′23.24"	159 m

### Water sampling and analytical methods

From February to September 2023, water samples were collected at nine selected sites (S1–S9) using the grab sampling method. The samples, taken 15–20 cm below the water surface, were stored in polyethylene bottles. Twenty-six physicochemical parameters were analyzed in triplicate in the laboratory, and mean values were recorded. Monthly data were categorized into monsoon (June–September) and non-monsoon (November-February) seasons for analysis. Physicochemical variables such as water temperature, pH, EC, and turbidity were measured using appropriate instruments at the sampling locations. The measurement of Total Dissolved Solids (TDS) was conducted employing cellulose nitrate membrane filters with a pore size of 0.45 μm. Alkalinity, total hardness, calcium, magnesium, DO, and BOD were assessed following standard methods^69^. COD was measured by the Winkler titration method^70^. Sodium and potassium were estimated with a UV–visible spectrophotometer (Systronic 2202). Analytical-grade chemicals were used without further purification. Calibration standards and reagents were prepared using deionized ultrapure water. Metal standards were created from a certified stock solution (1000 mg/L, Merck, Germany). For heavy metal (Al, As, B, Cd, Co, Cr, Cu, Fe, Mn, Ni, Pb and Zn) analysis, 250 mL water samples were filtered, acidified (pH < 2) with HNO3, and examined using ICP-OES (Nano ZS, Perkin Elmer, USA). All analyses were conducted at the Environmental Engineering Lab, BIT Mesra, Jharkhand, ensuring accuracy and repeatability (< 2%)^71^.

Measures were taken to prevent contamination and enhance the confidence of data for bias and variability. All apparatus and glassware were washed for 24 h with 10% HCl and rinsed twice with double-deionized water^72^. The chemical solutions were prepared from Merck-GR grade chemicals and reagents using double-deionized water. For calibrating instruments to obtain reliable results, bank samples were prepared from their stock solutions for each heavy metal parameter. The samples were analysed three times and instrumental calibration was done with drift samples for every 5 samples to ensure accuracy and efficiency for all metal analyses^73^. The reference materials provided by the American Public Health Association (APHA) were strictly followed. The uncertainty error was less than 10% for each heavy metal parameter analysed.

### Water quality indices

#### Overall index of pollution (OIP)

To compute the single-factor pollution index, the Overall Index of Pollution (OIP), as proposed by Ref.^32^, was employed. This index provided an assessment of the water's health status in Indian circumstances, and its calculation was executed using Eq. (1).

1 $${\text{OIP}}= \frac{1}{{\text{n}}} \sum_{{\text{i}}=1}^{{\text{n}}}{{\text{P}}}_{{\text{i}}}$$

where P_i_ = pollution index for the ith parameters and n = number of parameters.

2 $${{\text{P}}}_{\mathrm{i }}=\frac{\mathrm{Vn }(\mathrm{Observed \,value \,of\, the\, parameter})}{\mathrm{Vs }(\mathrm{Standard\, value\, of\, the\, parameter})}$$

Reference^32^ classified the water quality into five groups according to Table 2's OIP score. A score of less than 1.9 indicates exceptional water quality according to the OIP classification, placing it in Class C1. Class C2 water quality is considered satisfactory if the OIP score is less than 3.9. Less than 7.9, between 7.9 and 15.9, and more than 16 points to moderately (Class C3), moderately (Class C4), and severely (Class C5) contaminated situations, respectively.

**Table 2:** Classification of water quality in the overall index of pollution.

			Water quality parameters (limit/range)				
Water quality status	Class	Class Index (OIP Score)	pH	Hardness (mg/l)	Turbidity	BOD (mg/l)	TDS (mg/l)
Excellent	C1	1	6.5–7.5	75	5	1.5	500
Acceptable	C2	2	6.0–6.5 and 7.5–8.0	150	10	3	1500
Slightly polluted	C3	4	5.0–6.0 and 8.0–9.0	300	100	6	2100
Polluted	C4	8	4.5–5 and 9–9.5	500	250	12	3000
Highly polluted	C5	16	< 4.5 and > 9.5	> 500	> 250	24	> 3000

#### Nemerow’s pollution index (NPI)

Nemerow Pollution Index (NPI) was also used for the calculation of the Water Quality Index (WQI) which is a multi-element pollution index, one of the most widely accepted and increasingly used in recent years by researchers. NPI gives values about the range of pollution for individual water quality parameters concerning its standard value. NPI values also help to identify pollutants region; which is a piece of vital evidence concerning the worsening water quality of the area^74^.

The calculation method of the comprehensive pollution index is as follows:

3 $${P}_{N}=\sqrt{\frac{{(P1)}^{2}+{P}_{imax}^{2}}{2}}.$$

In the formula, ${P}_{N}$ is the comprehensive pollution index of the sampling point; ${P}_{imax}$ is the maximum value of the single-item pollution index of the pollutants at the sampling point; ${P}_{1}=\frac{1}{n}{\sum }_{i=1}^{n}$; P_i_ is the average value of the single-factor index.

#### Heavy metal pollution index (HPI)

HPI, as introduced by Refs.^48,75–78^, serves as a singular numerical representation that evaluates the collective impact of each chemical parameter on the overall pollution of heavy metals in a water body. It facilitates the assessment and scrutiny of the cumulative influence of all heavy metals on the comprehensive water quality. Essentially, this method employs a weighted arithmetic mean approach, assigning weights to specific heavy metals based on their significance and criticality about human and river health. The computation of the Heavy Metal Pollution Index (HPI) encompasses all analyzed heavy metals and their adherence to BIS water quality standards. The formula for calculating HPI is as follows:

4 $$HPI= \frac{{\sum }_{i=1}^{n}WiQi}{{\sum }_{i=1}^{n}Wi}.$$

In this equation, W_i_ represents the unit weight designated for each heavy metal, and Q_i_ denotes the sub-index of the ith heavy metal. The computation of W_i_ is derived from the subsequent equation:

5 $$Wi=\frac{k}{Vs}$$

In this context, k signifies the constant of proportionality (with k set to 1), and S_i_ stands for the recommended standard value for the ith heavy metal. The variable W_i_ spans from 0 to 1. The sub-index (Q_i_) value is calculated using the subsequent equation:

6 $$Qi=\left(\frac{Va-Vi}{Vs-Vi}\right)\times 100.$$

In this equation, V_a_ represents the value obtained through laboratory analysis, V_i_ is the ideal value, and V_s_ is the standard value of the ith heavy metal.

### Statistical analysis

Initially, basic statistical measures such as mean, minimum, and maximum values were computed using MS Excel 2016. Subsequently, principal component analysis (PCA) was applied to the Danro River and its various physicochemical parameters, including heavy-metal contents, aiming to identify potential sources of origin.

PCA serves as a multivariate statistical technique employed for data reduction, particularly valuable in hydrological studies for evaluating hydrochemical and hydrogeological parameters^79–81^. It assists in pinpointing the most influential parameters that collectively account for variability in a comprehensive dataset. By transforming correlated variables into a new set termed principal component, PCA effectively reduces the dimensions of the dataset. To enhance result accuracy, variable rotation using the varimax option was chosen^82^. This technique pinpoints the origins or contributing elements of fluctuations in water quality metrics. Because PCA offers a more precise identification of significant pollution components in the river parameters, it is superior to other approaches^83–85^. IBM Statistical Package for the Social Sciences (SPSS) version 27 was employed for statistical analysis, involving the bivariate correlation coefficient matrix and PCA extraction method. Pearson’s correlation coefficient method with a significance level of p < 0.05 was utilized. PCA was conducted using Kaiser’s varimax rotation principle, considering components with eigenvalues greater than 1.

### LULC classification

**Figure 3:**
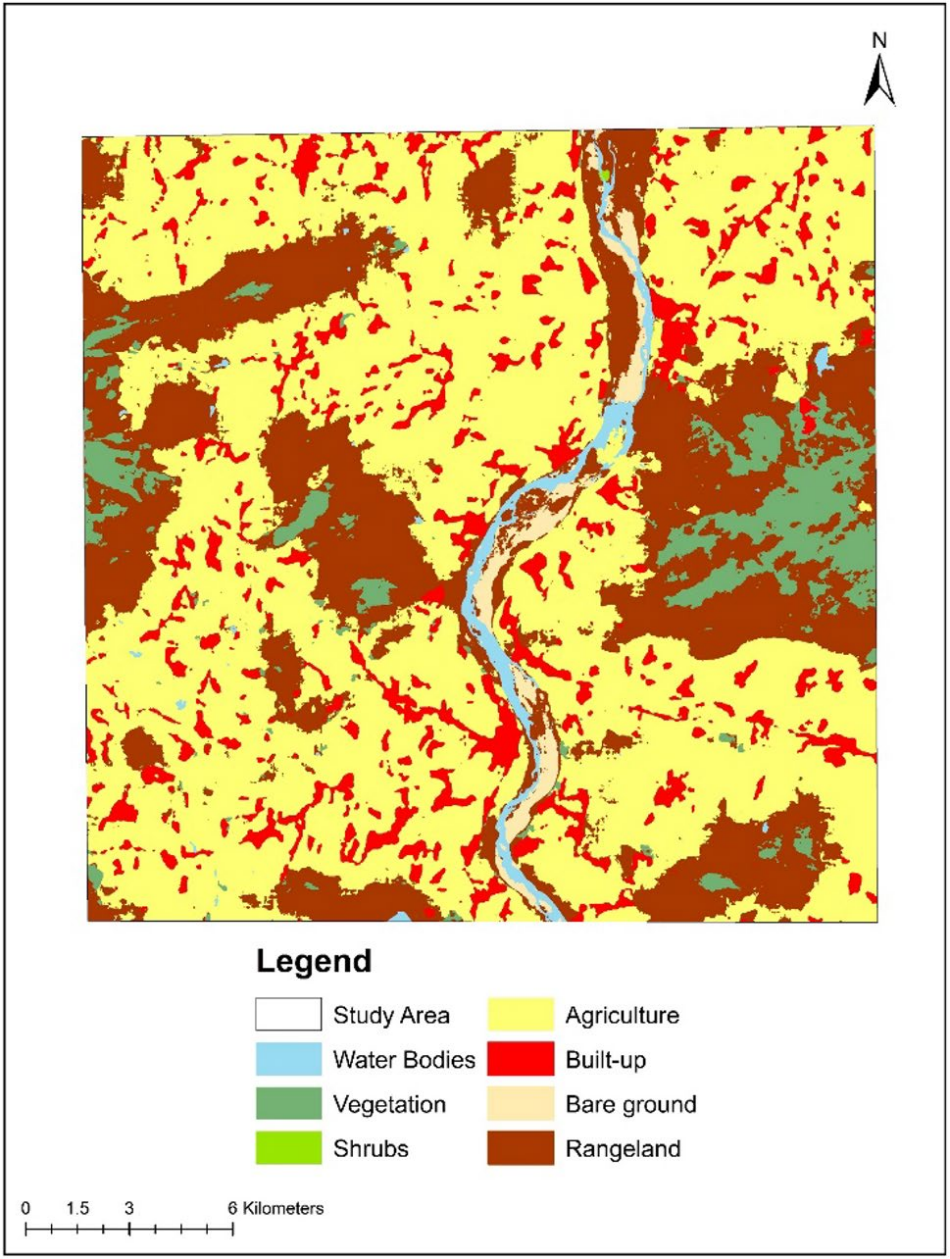
LULC classification map of the River Danro watershed (2024). Source: authors’ self-implementation with the ArcGIS software version 10.8 (http://www.esri.com).

Land use/land cover (LULC) classification entails the extraction of thematic details from satellite data related to diverse landscape features. Landsat-8 OLI data obtained on January 24, 2024, from the USGS Earth Explorer^86^, were utilized to create an LULC map for the study area, as illustrated in Fig. 3. The information obtained through LULC is beneficial for the efficient management and planning of land resources^87^. Despite the existence of various classification algorithms for satellite data, this study opted for the widely utilized Maximum Likelihood classification algorithm, implemented using ArcGIS 10.8 software. The study area was divided into seven classes—water body, vegetation, shrubs, agriculture, built-up, bare land, and rangeland. Results indicated that the majority of the study area is covered by water bodies (0.615%), followed by built-up areas (0.228%), bare land (0.089%), agriculture (0.027%), shrubs (0.022%), vegetation (0.018%), and rangeland (0.003%), respectively.

## Result and discussion

### Physico-chemical variables of Danro River

Table 3 presents a statistical overview of the analysed quality of river water. Water temperature, a critical parameter influencing biota activities, exhibited values ranging from 22.03 °C to 25.03 °C in non-monsoon and 22 °C to 24.77 °C in the monsoon season, with minimal seasonal variation. pH values were 8.53 and 8.10 for non-monsoon and monsoon seasons, respectively, indicating an alkaline nature at selected sites (S1–S9). Electrical conductivity (EC) showed concentrations of 122.53 μS/cm and 267.28 μS/cm for non-monsoon and monsoon seasons, indicating high ionic activity. Turbidity values exhibited variations, reaching a peak at 271.55 NTU during the monsoon and a minimum of 1.08 NTU in the non-monsoon season. Dissolved oxygen (DO) showed fluctuations within the range of 0.6 to 9.3 mg/l, with average values of 8.73 mg/l during the non-monsoon season and 1.10 mg/l in the monsoon. Biochemical oxygen demand (BOD) averaged 4.93 mg/l in non-monsoon and 5.52 mg/l during the monsoon. Chemical oxygen demand (COD) recorded a mean value of 122.22 mg/l. Alkalinity averaged 43.75 mg/l during the non-monsoon season and increased to 91.50 mg/l during the monsoon, while hardness displayed values of 133.3 mg/l and 708.33 mg/l, respectively. Total dissolved solids (TDS) remained within permissible limits; however, concentrations of calcium (Ca^2+^), magnesium (Mg^2+^), sodium (Na^+^), and potassium (K^+^) exceeded recommended levels, attributed to pollution from sewage and agricultural runoff. Elevated levels of total alkalinity, total hardness, and turbidity indicated human interventions and increased organic matter in the water.

**Table 3:** Descriptive statistics for water quality parameters of Danro River (Mean ± S.D.).

Parameters	Non-Monsoon	Monsoon
Temperature (°C)	23.79 ± 1.19	23.23 ± 1.00
pH	8.53 ± 0.51	8.1 ± 0.12
EC (µS/cm)	122.53 ± 4.41	267.28 ± 74.17
Turbidity (NTU)	1.076 ± 0.14	271.54 ± 16.93
TDS (mg/)	123.38 ± 1.62	231.57 ± 45.30
DO (mg/l)	8.73 ± 0.52	1.1 ± 0.65
BOD (mg/l)	4.93 ± 0.46	5.51 ± 0.49
COD (mg/l)	99.55 ± 20.39	144.9 ± 7.58
Sodium (mg/l)	3.31 ± 0.22	7.71 ± 0.50
Potassium (mg/l)	2.14 ± 0.25	4.23 ± 1.93
Total hardness (mg/l)	133.33 ± 24.83	708.33 ± 91.54
Alkalinity (mg/l)	43.75 ± 15.93	91.5 ± 5.99
Ca (mg/l)	62.06 ± 2.07	63.18 ± 0.58
Mg (mg/l)	18.25 ± 0.52	18.10 ± 0.39

### Description and comparison of the heavy metals with BIS guidelines

There were no substantial differences in heavy metal concentrations among the sampling stations. Mean values of heavy metals for River Danro are presented in Table 4, showcasing the following trend: Al > Fe > Mn > Cd > Cr > Cu > Zn > Ni > B > As > Pb, with the highest average value for Al and the lowest for Pb.

**Table 4:** Descriptive statistics for heavy metals in Danro River (Mean ± S.D.).

Heavy metals (mg/L)	Limit of detection (LOD) in mg/L	Mean ± S.D
Al	0.001	0.930 ± 0.832
As	0.001	− 0.047 ± 0.049
B	0.001	− 0.028 ± 0.002
Ca	0.1	27.143 ± 7.225
Cd	0.001	0.002 ± 0.001
Co	0.001	− 0.006 ± 0.002
Cr	0.001	− 0.009 ± 0.002
Cu	0.001	− 0.012 ± 0.001
Fe	0.001	0.573 ± 0.545
Mg	0.1	7.212 ± 1.752
K	0.1	3.364 ± 0.460
Mn	0.001	0.006 ± 0.011
Na	0.1	21.446 ± 8.045
Ni	0.001	− 0.021 ± 0.004
Pb	0.001	− 0.122 ± 0.023
Zn	0.001	− 0.018 ± 0.004

Negative values indicate Below Detection Limit (BDL).

The Al content in the Danro River exceeded the BIS limit for surface waters at all sampling sites. Nevertheless, the concentrations of Cu, Cr, Zn, Mn, B, Pb, and Ni remained within the maximum permissible limits as defined by the BIS (2012) standards. For As, concentrations exceeded the BIS (2012) guidelines for drinking water at sites S6 and S9, while Cd surpassed the BIS (2012) guidelines at sites S1, S2, and S6. Elevated Fe content exceeded permissible limits for drinking water at sites S4, S5, S6, S8, and S9. The concentrations of dissolved heavy metals at the remaining three sites were low, suggesting no additional sources of contamination near the river.

### Water quality analysis using overall index of pollution

The evaluation of water quality was carried out through the utilization of the Overall Index of Pollution (OIP), involving the scrutiny of 10 physicochemical parameters: pH, electrical conductivity, turbidity, total dissolved solids (TDS), dissolved oxygen (DO), biochemical oxygen demand (BOD), total hardness, calcium, magnesium, and alkalinity. This analysis was carried out at specific sites along the Danro River during both non-monsoon and monsoon seasons. The OIP scores highlighted that electrical conductivity, turbidity, and total hardness were the predominant parameters at all the sampling sites. The summarized OIP values for water samples from six selected sites during each season are presented in Table 5. The overall findings revealed an excellent water quality status (1 < OIP < 1.9) during the non-monsoon season and slightly polluted conditions (4 < OIP < 7.9) during the monsoon season in the Danro River.

**Table 5:** Summary of Overall Index of Pollution (OIP) values of the Danro River.

Sampling site	Non-Monsoon			Monsoon		
	OIP	Class	Water quality status	OIP	Class	Water quality status
S1	0.734	C1	Excellent	6.156	C3	Slightly polluted
S2	0.683	C1	Excellent	6.835	C3	Slightly polluted
S3	0.715	C1	Excellent	6.622	C3	Slightly polluted
S4	0.714	C1	Excellent	6.035	C3	Slightly polluted
S5	0.722	C1	Excellent	5.715	C3	Slightly polluted
S6	0.729	C1	Excellent	6.319	C3	Slightly polluted
Average	0.716	C1	Excellent	6.280	C3	Slightly polluted

Based on the OIP index scores, the health status during the non-monsoon season was excellent at all sampling sites (S1: 0.734, S2: 0.683, S3: 0.715, S4: 0.714, S5: 0.722, S6: 0.729). Conversely, the monsoon season recorded higher OIP values ranging from 5.715 at S5 to 6.835 at S2, with an average value of 6.280, indicating a slightly polluted water quality. This could be attributed to significant sediment runoff, bank erosion, the convergence of waters from various points (sewage water discharge), and non-point sources (agricultural runoff) during the monsoon season.

The overall assessment classified the water quality of the Danro River as Class C1 during the non-monsoon season and Class C3 during the monsoon season across all sampling sites. Temporary turbidity was notably elevated at all sites, attributed to sediment removal from the riverbed, resulting in increased parameters such as TDS, conductivity, and hardness. A similar study by Ref.^88^ on the Ganga River, utilizing the OIP index, revealed acceptable water quality in summer and winter, with pollution observed in the monsoon season due to significant sediment runoff, debris, and bank erosion caused by elevated stream flow.

### Water quality analysis using Nemerow pollution index

Based on Eq. (3), the Nemerow Pollution Index values were computed and are presented in Table 6. A lower Nemerow Pollution Index value indicates higher water quality. The assessment results reveal that the water quality pollution index for the Danro River ranged between 0.10 and 1.74 during the non-monsoon season and between 0.22 and 27.15 in the monsoon season. Generally, the water exhibits acceptable quality, with pH levels close to the standard during both seasons. However, turbidity significantly increases during the monsoon, indicating potential pollution sources. Conductivity remains within acceptable limits for both seasons. Dissolved oxygen levels, though generally acceptable, decrease notably during the monsoon. Biochemical Oxygen Demand is slightly higher during the monsoon, suggesting increased organic load. Total Dissolved Solids, alkalinity, calcium, and magnesium levels are within standards, indicating overall good water quality. Total Hardness exceeds the standard during the monsoon, highlighting a potential concern. These results emphasize the need for targeted interventions, especially during the monsoon, to address specific parameters and maintain a consistently high water quality standard.

**Table 6:** Numero’s Pollution Index (NPI) values of the Danro River.

Sl. no	Parameters	Standard	NPI values (Non-Monsoon)	NPI values (Monsoon)
1	pH	8.5	1.004	0.953
2	Turbidity, NTU	10	0.108	**27.155**
3	Conductivity, µs/cm	300	0.408	0.891
4	Dissolved oxygen, mg/L, Min	5	**1.746**	0.220
5	Biochemical oxygen demand, mg/L, Max	5	0.986	**1.104**
6	Total dissolved solids, mg/L, Max	500	0.247	0.463
7	Alkalinity	120	0.365	0.763
8	Total hardness, mg/L	300	0.444	**2.361**
9	Calcium (as Ca), mg/L	75	0.560	0.842
10	Magnesium (as Mg), mg/L	30	0.667	0.603

Significant values are in bold.

The outcomes derived from the Nemerow Pollution Index (NPI) and the Overall Index of Pollution (OIP) contribute complementary perspectives on water quality. The NPI values for specific parameters offer a nuanced insight into the distinct factors influencing water quality, whereas the OIP provides a comprehensive overview by assigning an overall pollution index. By comparing the two indices, it becomes evident that the monsoon season has a notable impact on overall water quality, as both indices show an increase in pollution levels during this period. This correlation suggests a potential link between the individual parameters measured by the NPI and the overall water quality status reflected in the OIP.

### HPI index

The Heavy Metal Pollution Index (HPI) was computed individually for each sampling location to compare pollution loads and evaluate water quality at the selected stations and during different seasons (refer to Table 7). These values represent the cumulative impact of various heavy metals. According to the guidelines for drinking water by BIS (2012)^89^, the HPI findings suggest that the surface water bodies investigated in our study are extensively contaminated by heavy metals and are unsuitable for potable purposes, with HPI values surpassing 100.

**Table 7:** Heavy Metal Pollution Index (HPI) values of the Danro River.

Site	HMI value
1	245.9
2	202.9
3	316.4
4	330.4
5	256.9
6	331.0
7	267.9
8	307.0
9	329.6

Noteworthy variations in HPI values are evident across different sampling sites. Furthermore, the mean HPI values for each sampling site indicate that the pollution load is most pronounced at sampling site 6 (HPI 331.04). Elevated HPI values are attributed to industrial wastewater, domestic sewage, landfill leachate, and agricultural runoff. Consequently, it is affirmed that water pollution poses a significant concern, yet no solutions have been proposed as of now.

### Principal component analysis

Principal Component Analysis (PCA) was conducted on the correlation matrix of rearranged data for the Danro River. The variance/covariance and factor loadings of variables with eigenvalues were calculated. A combination of correlation matrix, factor analysis, and cluster analysis was employed to evaluate contamination levels, identify chemical processes, and trace diffusion paths. Varimax rotated factor analyses were performed on 21 parameters from the PCA, and factor loadings were computed. Four major components, with eigenvalues exceeding one, were selected, explaining 92.34% of the total variance. Features with factor loadings greater than 0.5 were considered significant for interpreting each component. Communalities for all metals ranged between 0.87 and 1, indicating satisfactory allocation to identified factors. The physical interpretation of each factor or source was based on its association with the strong loading of marker elements typically emitted from that source. The first component (factor 1), associated with pH, EC, DO, BOD, Ca, Mg, Cd, Pb, and turbidity, explained 41.23% of the total variance, indicating the presence of high organic content in the water and pollution from electrical conductivity due to riverbank erosion from dredging activities. Elevated levels of Pb were linked to highways, road dust, traffic activities, and major roads^90–92^. The second component (factor 2), primarily linked to TDS, DO, Cr, Mn, and Zn, explained 21.78% of the total variance, suggesting possible industrial discharges. Elevated concentrations of Cr, Mn, and Zn may indicate activities such as metal manufacturing, electroplating, and mining, releasing metals into the water^93^. High TDS levels were associated with agricultural runoff, especially in areas with fertilizer and pesticide use, while urban areas contributed to increased TDS and metal levels in surface water through stormwater runoff. The third component (factor 3), including B and Ni, explained 12.36% of the total variance, suggesting potential sources like industrial discharges, agricultural runoff, mining activities, urban stormwater runoff, natural geological processes, wastewater treatment plant effluents, atmospheric deposition, and waste disposal sites near the river^94^. The fourth component (factor 4), associated with turbidity, Al, Cd, and Fe, explained 11.07% of the total variance, indicating influences from industrial discharges, agricultural runoff, natural weathering, urban runoff, mining activities, wastewater discharges, and atmospheric deposition^95–97^. Ultimately, the fifth component (factor 5), primarily linked to Cr, elucidated 5.88% of the total variance, indicating inputs from human activities, particularly agricultural practices such as the application of pesticides and fertilizers, along with lithogenic sources^98,99^.

### Land use change and WQ

The studied area has undergone significant changes in land use, primarily driven by public activities such as deforestation, construction, and cultivation. This dynamic land use transformation was overlaid and correlated with the Water Quality Index (WQI) to assess the extent to which human activities contributed to the degradation of river water quality, as depicted in Fig. 3. Land use changes exert diverse impacts on local temperature, natural ecosystems, socio-economic factors, and policy formulation and implementation^100–102^. Numerous studies have highlighted the connection between land use changes and seasonal variations in water quality^103–108^. Water quality exhibits variations between the non-monsoon and monsoon seasons, providing direct evidence of the influence of anthropogenic activities. The quality of water is poorer during the monsoon season due to increased river flow, making it more susceptible to non-point source pollutants mobilized by the higher velocity of the river water during this period^109^. Physicochemical analysis of water parameters revealed unsatisfactory water quality in areas with extensive human intervention. Natural forest cover serves as a nutrient retention system, fostering a biologically rich environment conducive to water and aquatic life^110^. Conversely, areas heavily impacted by human activities exhibit adverse effects^111,112^. Additionally, the disruption of natural systems and land use changes significantly contribute to total dissolved solids, nitrogen and phosphorous deposition, influenced by both point and non-point source pollution^113^. High concentrations of Mg2 + , Na + , K + , Cl − , F − , and Fe2 + suggest the discharge of significant amounts of untreated sewage and agricultural waste into the river at the study sites. This aligns with previous studies indicating that water quality is substantially influenced by untreated waste^114–117^. The predominant factor influencing the presence of heavy metals in water samples is associated with the land use pattern. Specifically, built-up areas, concentrated in the southern part of the study area, exhibit a notable correlation with the composition of heavy metals in water samples. These built-up areas can act as non-point sources of heavy metals due to diverse activities, including small-scale industries (such as leather and textile) and human settlements where the discharge of wastewater introduces various heavy metals like Fe, Zn, Mn, etc., into river water bodies. Simultaneously, activities like sand mining, natural factors like rock weathering, and other domestic effluents in the upstream region further contribute to elevated concentrations of heavy metals like Ti, Cu, Cr, Ni^118^. The mean Heavy Metal Pollution Index (HPI) was found to be above 100, rendering the water unsuitable for human use and irrigation, as contamination can propagate through the food chain, causing long-term health issues. Table 8 outlines the contaminants of concern, their sources, impacts, and management techniques.

**Table 8:** Contaminants of concern, their source, impact and management techniques.

Contaminant of concern (sampling locations)	Observed LULC	Natural sources	Anthropogenic sources	Adverse effects on humans	Ecological impact	Technical solution	Ecological solution (phytoremediation)
Al (S1-S9)	Agriculture and semi-urban area with Danro River	Acid rain, Acidic rocks and soils^119–121^	Infiltration of wastewater from towns^122^	Loss of memory, severe trembling, dementia^123^	Habitat disruption, Greenhouse gas emissions, air and water pollution^124,125^	Active carbon absorbtion^126,127^	Water Hyacinth, Moringa oleifera seeds and Boscia senegelensis seeds^128,129^
Ca (S1-S9)	Agriculture and semi-urban area with Danro River	Limestone, dolomite, gypsum, and other calcium-containing rocks and minerals^130^	Originates from the weathering of carbonate rocks^131^	Stomach upset, nausea, vomiting and constipation^132,133^	Making things from calcium, like limestone, can harm nature due to changes in habitats and using a lot of energy, depending on how it's done^134^	Reverse Osmosis^135^	Schoenoplectus litoralis and Hordeum vulgare^136–138^
Cd (S1, S2, S6)	Covering rural built-up and mainly crop grown region	Volcanic eruptions, weathering, natural fires, and dust storms^139,140^	Welding, electroplating, pesticides, fertilizer, batteries, nuclear fission plant^141,142^	Psychological disorders, diarrhoea and damage of immune system^143^	Soil and water pollution, bioaccumulation in organisms, disruption of aquatic ecosystems, and potential threats to human health through the food chain^144,145^	Nanocomposite adsorbents^146^	Water Hyacinth, Cattail^147,148^
Fe (S4, S5, S6, S8, S9)	Rural built-up and agriculture-dominated area	Mafic rocks, limestones and shales^149^	Erosion of soil, runoff from agricultural field, construction sites, deforested area, urban runoff and decaying organic matter^150^	Staining of cloths and plumbing material^151^	Habitat disruption, water and air pollution, and significant ecological impacts on landscapes and ecosystems^152^	Chemical oxidation, filtration^127^	Mango leaf, guava leaf, Typha domingensis and duckweed Lemna minor^128^
Mg (S1-S9)	Agriculture and semi-urban area with Danro River	Mafic rocks, limestones and shales^153^	Fertilizer, cattle feed^154,155^	Diarrhea that can be accompanied by nausea and abdominal cramping^133,156^	Making things from magnesium, like limestone, can harm nature due to changes in habitats and using a lot of energy, depending on how it's done^157,158^	Water softening^159^	Schoenoplectus litoralis and Hordeum vulgare^138^
K (S1-S9)	Agriculture and semi-urban area with Danro River	Weathering of rocks^160,161^	Municipal and industrial sewage discharges and agricultural runoff^162^	Heart palpitations, shortness of breath, chest pain, nausea, or vomiting^163^	Natural potassium is eco-friendly, but environmental issues arise from the extraction and use of potassium-based fertilizers, causing habitat disruption, energy use, and possible water pollution, depending on industrial practices^164^	Coagulation^165^	Azolla caroliniana^137^
Na (S1-S9)	Agriculture and semi-urban area with Danro River	Precipitation and weathering of silicate minerals^166^	Road salt, water treatment chemicals, domestic water softeners, and sewage effluents^167^	Excessive thirst, bloating and blood pressure rise^168,169^	Natural sodium is generally eco-friendly, but the environmental impact of extracting and using sodium compounds, like table salt, may lead to soil salinity, water pollution, and harm to aquatic ecosystems, depending on industrial practices^170^	Reverse osmosis^171,172^	Schoenoplectus litoralis and Hordeum vulgare^173,174^
As (S6, S9)	Rural built-up and agriculture-dominated area	Sulphide mineral deposits and sedimentary deposits deriving from volcanic rocks^175–177^	Pesticides, fungicides, metal smelters, mining and burning of fossil fuel^176,178^	Kidney, skin, blood and Liver disorders^179,180^	Soil and water contamination, bioaccumulation in food chains, and detrimental effects on aquatic life, with long-term persistence and potential human health hazards^181,182^	Oxidation, Coagulation, precipitation, filtration, Adsorption, Ion exchange and Membrane techniques^183–187^	Duckweed (Lemna minor L), water hyacinth (Eichhornia crassipes), water zinnia (Wedela trilobata Hitchc.) and water lettuce (Pistia stratiotes L.)^188^
Pb (S1-S9)	Agriculture and semi-urban area with Danro River	Soil erosion, volcanic eruptions, sea sprays, and bush fires^189^	Paint, pesticides, batteries, automobile emission, mining, and burning of coal^189–191^	Lead toxicity leads to anaemia, nervous system. Gastro-intestinal respiratory and cardiovascular diseases^192^	Soil and water contamination and lead-based products, adverse effects on wildlife and plant life, potential bioaccumulation in food chains, and risks to human health^193,194^	Sediment dredging^195^	Water Hyacinth, Cattail^196^

### Treatment of river water contaminated with heavy metals

The intricate composition of wastewater, influenced by numerous coexisting compounds, poses a challenge for current technologies to precisely recognize detailed compositions. Early investigations relied on physicochemical tests like complexometric titration, ion exchange, and stripping voltammetry to evaluate complexation features, hindering the determination of exact coordination conditions of heavy metals^197–199^. A promising solution to address pollutants in rivers and water bodies, detrimental to marine life and human health, is the eco-friendly and cost-effective approach of phytoremediation. This method involves using plants such as Water Hyacinth, Indian Mustard, Sunflower, Vetiver Grass, Azolla, Neem Tree, Bamboo, and Spider Lily to absorb, accumulate, and detoxify heavy metals from water. In India, where water pollution is a significant concern, these plants are employed for their ability to remediate heavy metals effectively. The effectiveness of phytoremediation depends on factors like specific contaminants, environmental conditions, and plant species used, and it is often used in combination with other remediation techniques for optimal results. Researchers globally, including^200,201^, have studied bioremediation of heavy metals.

**Figure 4:**
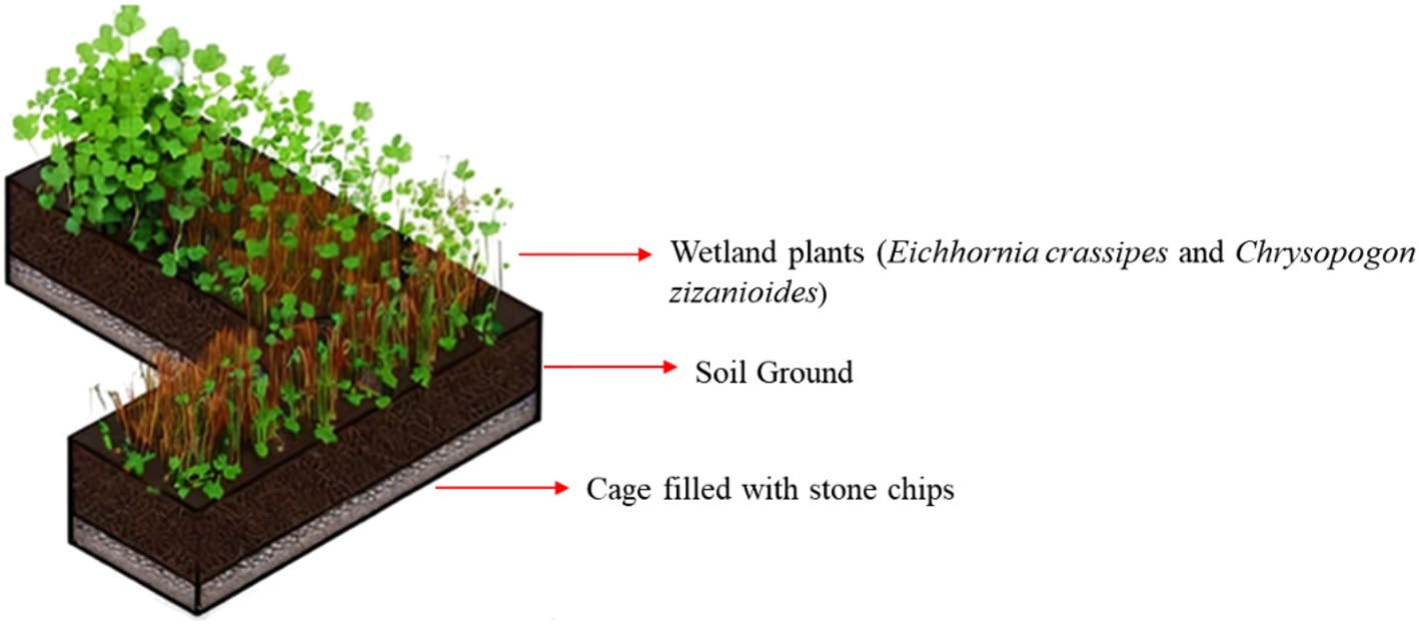
The view of the formation layers of the “L” shaped design model.

The present work focuses on evaluating wastewater remediation using constructed riverbeds containing *Eichhornia crassipes* (Water hyacinth) and *Chrysopogon zizanioides* (Vetiver grass), which are found locally. Within the study's scope, there was a need for a cost-effective, portable, and maintenance-free design model with no energy requirements. The design, shaped like the letter "L" serves as the basis for applying the phytoremediation method. The dimensions can be adjusted based on the river's structure, offering flexibility in response to factors such as wastewater discharge direction and areas with high pollution levels. The "L" form allows for diverse design combinations, adapting to variations in stream conditions or facilitating community use. The primary structure comprises a 5 × 5 wire cage filled successively with stone chips and a soil layer, covered by a 15 × 15 wire on top. The stone chips layer adds weight and diminishes surface water flow in the stream. The soil layer, crucial for plant development, accumulates water where plant roots are situated, enabling the extraction of heavy metals from the water while releasing oxygen. The finalized system is illustrated in Fig. 4. Plant species like *Typha latifolia* and *Monochoria hastata*, which are again native plant species in Jharkhand, could be used in the riparian zones along the riverbed to hold additional pollutants. The treatment efficiency for these species has been recorded by various authors and is discussed in Table 9.

**Table 9:** Phytoremediation Efficiency of Selected Plant Species in this study for Heavy Metals.

Selected species	Efficiency	Reference
*Eichhornia crassipes*	99.5% removal of heavy metals (such as Mn, Cd, Fe, Zn, Cu, As, and Pb) and plant nutrients (such as N, P, K, Ca, Mg)	^200,202–207^
*Chrysopogon zizanioides*	95% removal of N, P, Zn, Mn and Ni	^208–215^
*Monochoria hastata*	Cd can be classified as moderate accumulator	^216–222^
*Typha latifolia*	Potential to remove both salts (Na, Cl, Ca, Mg) and heavy metals (Zn, Cu, Fe, Mn)	^223,224,224–227^

**Figure 5:**
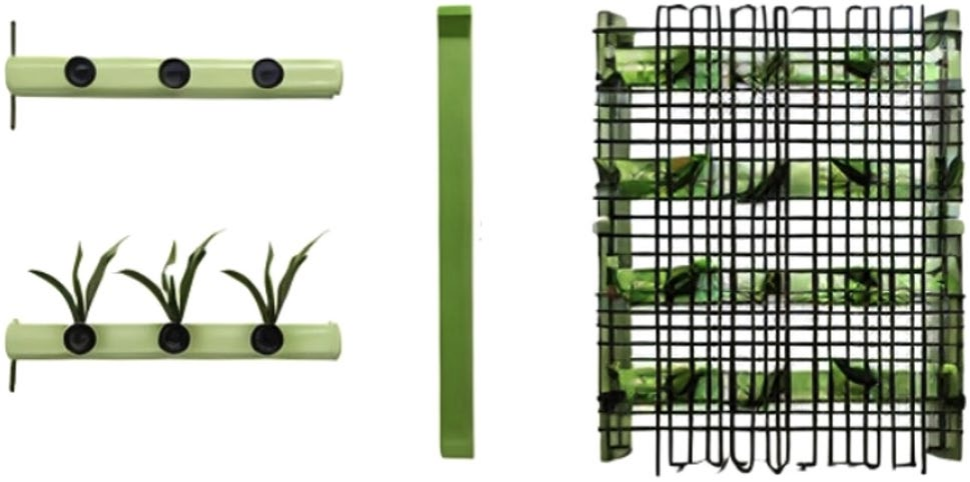
Plants Floating Bed.

An alternative approach to address urban river pollution, stemming from primary pollutant sources and their impacts on soil, water, and living organisms, involves the use of ecological floating beds (see Fig. 5). The structure of the Plants Floating Bed is constructed using two types of thick bamboo tubes (TBT). The first type of TBT requires perforations at regular intervals for transplanting plants into the upper holes. The second type of TBT, without holes, is utilized to secure the perforated TBT and provide buoyancy. These two types of TBT are combined and fastened together, with a net drawn over them. *Eichhonia crassipes* and *Chrysopogon zizanioides* could be planted on the floating bed in turn.

### Limitations and future research needs

There are currently some drawbacks to using existing methods for evaluating water quality. Firstly, most assessments today only look at one thing at a time, instead of using a more comprehensive approach. Scientists have mainly focused on changes in pollutant levels and water properties, but not the whole picture. This can lead to unconvincing results, which doesn't help manage projects or improve how well cleanups work (as reported by Rohr et al. 2016). There also aren't enough examples of how these assessments work in real-world situations, which are often more complex. Secondly, even though indexes help understand how bad heavy metal contamination is and its impact, they have some weaknesses. These indexes rely on calculations based on things like how much metal is present and how it compares to natural levels in the environment, as well as the type of sample being tested (water vs. sediment). This simplified approach doesn't capture the full complexity of how heavy metal pollution works in real-world settings. In other words, a single index can't give the whole picture. Additionally, the lack of reliable background data for different locations can make the results inaccurate. Choosing which standards to use can further confuse things for those making decisions.

Based on these gaps and challenges, future research is recommended as follows:

1. Develop clear standards for both water quality and remediation techniques. Before that can happen, though, there needs to be a standard way to analyze pollutants and assess the ecological effects of contamination.
2. Create a comprehensive assessment system that uses these established standards. This means figuring out how the different parts of a remediation evaluation fit together.
3. Design specific assessment methods for cleaning up contaminated water on-site. These methods should consider the type of pollutant being addressed and what the cleaned-up water will be used for. For instance, if the water will be used for farming, researchers would need to assess the potential health risks.
4. Investigate how assessment methods, water treatment technologies, and cost factors work together in real-world settings. This will ensure that the assessment methods are practical.
5. Develop better ways to measure natural background levels of heavy metals in different areas. This will provide more accurate data for the index calculations, leading to more precise assessments of pollution and risk for each location.
6. Continuously research and update standards, regulations, and toxicity values for heavy metals. This will improve the overall reliability of using indexes to assess heavy metal pollution.
7. Focus on creating new, multi-purpose indexes that can handle a wider range of situations. Ideally, these new indexes could assess the risks and pollution levels of multiple contaminants in different environments (water, soil, air).
8. As computer science advances, integrating these indexes with models is a promising approach. This could help reduce errors during calculations and save time.

## Conclusion

In conclusion, our in-depth analysis of River Danro’s physicochemical variables and heavy metal concentrations paints a detailed picture of its environmental health. The physicochemical parameters, including water temperature, pH, electrical conductivity, turbidity, dissolved oxygen, biochemical oxygen demand, and others, exhibit notable variations between non-monsoon and monsoon seasons. Elevated levels of calcium, magnesium, sodium, and potassium indicate pollution from sewage and agricultural runoff, impacting the river's overall water quality. Heavy metal concentrations, while not significantly different between sampling stations, reveal concerning levels of aluminium exceeding BIS guidelines at every site. Other heavy metals such as copper, chromium, zinc, manganese, boron, lead, and nickel fall within permissible limits, with arsenic and cadmium surpassing guidelines at specific sites. Iron concentrations exceed limits at select sites, emphasizing potential sources of contamination. The Overall Index of Pollution (OIP) scores highlight electrical conductivity, turbidity, and total hardness as the most influential parameters, categorizing Danro River's water quality as excellent in non-monsoon and slightly polluted in monsoon seasons. Nemerow Pollution Index (NPI) values further corroborate this, revealing acceptable water quality with occasional spikes in pollution during the monsoon. The Heavy Metal Pollution Index (HPI) indicates significant contamination, especially at site 6, attributed to industrial wastewater, domestic sewage, landfill leachate, and agricultural runoffs. Principal Component Analysis (PCA) identifies four major components, associating specific parameters with potential pollution sources, and emphasizing the need for targeted interventions. Land use changes, primarily due to deforestation, construction, and cultivation, correlate with water quality deterioration. Anthropogenic activities during the monsoon season exacerbate water pollution, with high-flow conditions contributing to non-point source pollutants. In summary, the comprehensive assessment of River Danro's water quality using multiple indices, multivariate statistics, and geospatial analysis underscores the urgency for effective management strategies. The study provides valuable insights for policymakers and environmentalists to formulate targeted interventions, ensuring the sustainability and health of this vital water resource. Immediate regulatory actions and proactive measures are essential to address the identified pollution sources and safeguard the long-term well-being of River Danro.

The recommendations mentioned in the above sections could prove effective in addressing these issues:

(i) Our study's site-specific analysis of water quality indicates that the impact on water quality is influenced by agricultural land use and human activities. Thus, targeted efforts for water restoration in key areas are crucial for enhancing overall water quality.
(ii) Vegetation acts as the natural mechanism of nutrient and pollutant uptake. Thus, increasing the vegetation in the riparian zones of Danro River is suggested as the natural barrier for removing the contamination.
(iii) Increased public awareness, concern, and active involvement at the local level are essential to conserve water resources and enhance overall quality of life.
(iv) Lastly, floating bed remediation could be a promising approach for mitigating the impacts of sand mining and reducing the pollutant load on aquatic ecosystems. By restoring habitat, improving water quality, and preventing erosion, floating beds help promote ecological resilience and support the long-term health of freshwater ecosystems.

## Data Availability

Satellite Data has been obtained from the USGS portal. Rest all is primary data collection and analysis. No plant was used in the study for the experiment. The data that support the findings of the study are available from the corresponding author upon reasonable request.
